# Micro-Patterning of PEG-Based Hydrogels With Gold Nanoparticles Using a Reactive Micro-Contact-Printing Approach

**DOI:** 10.3389/fchem.2018.00667

**Published:** 2019-01-17

**Authors:** Cigdem Yesildag, Zhaofei Ouyang, Zhenfang Zhang, Marga C. Lensen

**Affiliations:** Nanopatterned Biomaterials, Department of Chemistry, Technische Universität Berlin, Berlin, Germany

**Keywords:** PEG hydrogel, Au NPs, micropatteming, cell adhension, multifunctional

## Abstract

In this work a novel, relatively simple, and fast method for patterning of gold nanoparticles (Au NPs) on poly(ethylene glycol) (PEG)-based hydrogels is presented. In the hereby exploited reactive micro-contact printing (reactive-μ-CP) process, the surface of a micro-relief patterned PDMS-stamp is first functionalized with an amino-silane self-assembled monolayer (SAM), which is then inked with Au NPs. The stamp is subsequently brought into conformal contact with thiol-functionalized PEG-based hydrogel films. Due to the strong gold-thiol interactions the Au NPs are adequately and easily transferred onto the surfaces of these soft, multifunctional PEG hydrogels. In this way, defined μ-patterns of Au NPs on PEG hydrogels are achieved. These Au NPs patterns allow specific biomolecular interactions on PEG surfaces, and cell adhesion has been studied. Cells were found to effectively adhere only on Au NPs micro-patterns and to avoid the anti-adhesive PEG background. Besides the cell adhesion studies, these Au NPs μ-patterns can be potentially applied as biosensors in plasmon-based spectroscopic devices or in medicine, e.g., for drug delivery systems or photothermal therapies.

## Introduction

Hydrogels are three-dimensional networks of hydrophilic polymers, which can imbibe high amounts of water relative to their own weight and are widely used in industry as care products, agriculture, biology, and medicine (Drury and Mooney, 2003; Peppas and Hoffman, 2013). Recent research fields are focused on applying specific functionality to the hydrogel (Haraguchi and Takehisa, 2002; Schexnailder and Schmidt, 2009; Gaharwar et al., 2015; Motealleh and Kehr, 2017; Ren et al., 2017a; Yesildag et al., 2018a). Notably multi-functional hydrogels are highly desired as biomaterials that simultaneously exhibit various beneficial properties (Tkachenko et al., 2003; Sukhorukov et al., 2007; Chen et al., 2013; Li et al., 2017).

Biomaterials are natural or synthetic materials that are designed to augment or replace tissues, organs, or functions of the body, in order to maintain or improve the quality of life of the individual (Boretos and Eden, 1984; Williams, 1987). Examples for biomaterials are implants (Davis, 2003; Saini et al., 2015), prosthesis (Davis, 2003), wound healing or tissue regeneration (Lee and Mooney, 2001; El-sherbiny and Yacoub, 2013), repairing or supporting materials (Seal et al., 2001; Davis, 2003), and also biochips (Ferrari et al., 2007; Vo-Dinh and Cullum, 2008; Veitinger et al., 2014) which can be used for example as biosensors (Ferrari et al., 2007; Lee, 2008; Vo-Dinh and Cullum, 2008; Shruthi et al., 2014; Sabr, 2016). For the successful design of biomaterials and the (micro- or nano-)fabrication of biointerfaces, fundamental understanding of interactions of the biomaterials with the biological system (e.g., organic tissue or bodily fluid) is required.

Precursors for hydrogels can have natural or synthetic origins. Whereas, hydrogels from natural origin are intrinsically cytocompatible, cytotoxicity can be an issue in the case of hydrogels from synthetic precursors. On the other hand, synthetic hydrogels may exhibit better mechanical integrity (bio)chemical stability, defined pore sizes, while the physical and chemical reactivity are easier to control. Among others, especially poly(ethylene glycol) (PEG) based hydrogels are highly desired in biological applications, due to the non-toxicity, non-immunogenicity, and bio-inert characteristics (Zalipsky and Harris, 1997). In our group, particularly PEG macro-monomers with acrylate or vinyl sulfone end-groups have been studied to have the ability to be cross-linked by UV-photo-polymerization process to achieve PEG hydrogels (see Table 1).

**Table 1:** Examples of PEG-based macromonomers for preparing hydrogels.

**Material**	**PEG Diacrylate (PEG)**	**8-arm PEG Acrylate (8PEG)**	**8-arm PEG Vinyl Sulfone (8PEG-VS)**
Structure	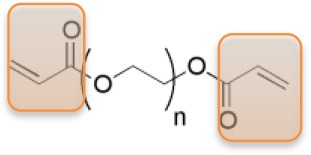	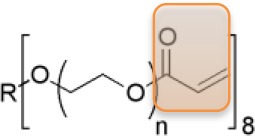	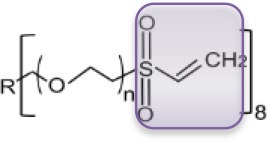
	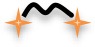	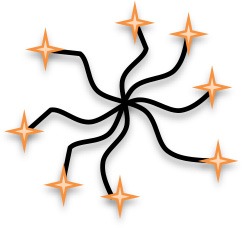	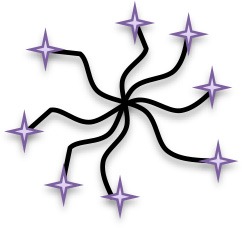
M_w_ [Da]	575	15k	15k
Chain length	n ~ 72	n ~ 40	n ~ 40
State at r.t.	Liquid	Solid	Solid
Gel formation	UV	UV or Michael type addition (degradable)	UV or Michael type addition (non-degradable)

**Figure 1:**
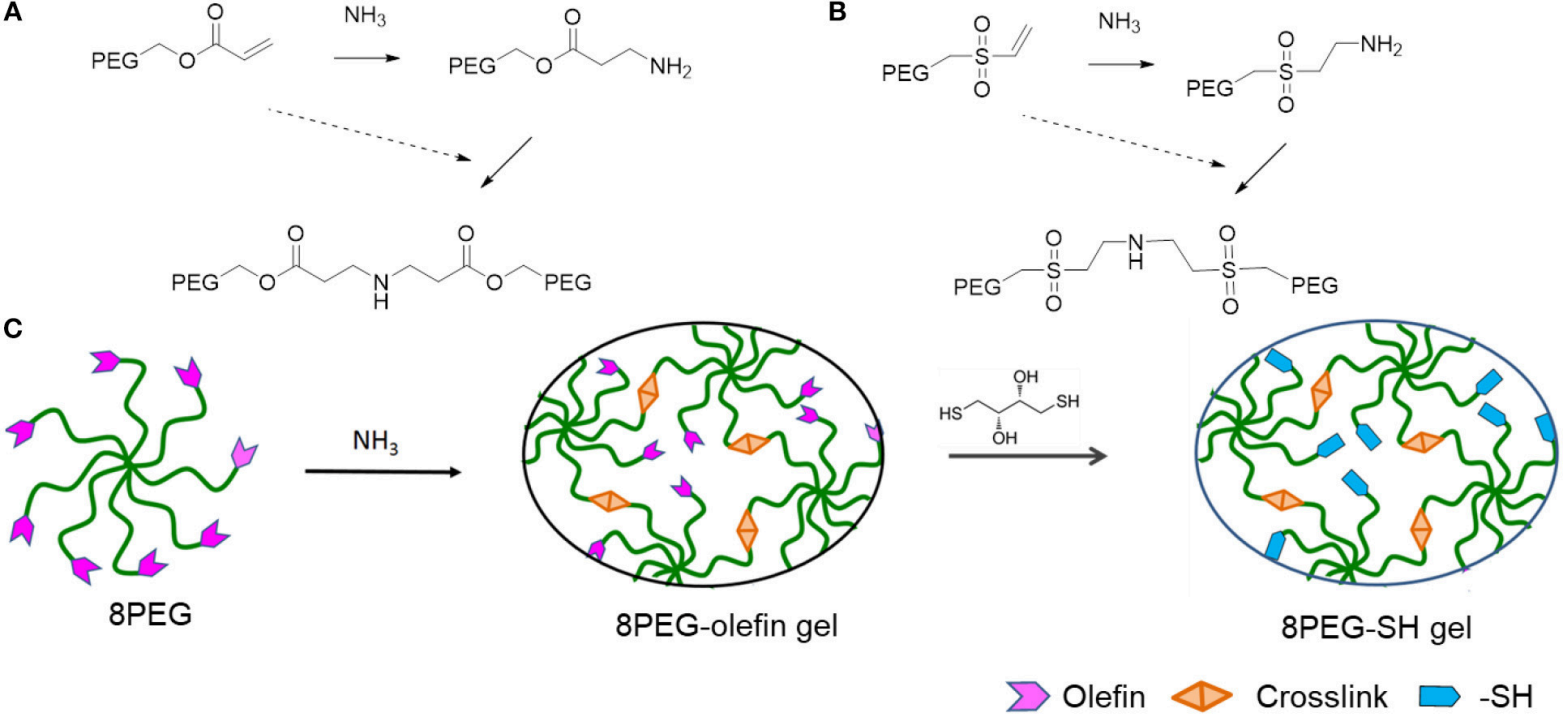
Schematic representation of PEG-based hydrogels prepared through amine-Michael type addition and modification via sulfur-Michael type addition. 8PEGacr **(A)** or 8PEGvs **(B)** based hydrogels prepared via amine-Michael type addition. **(C)** The preparation of both 8PEG-olefin and 8PEG-SH hydrogels. 8PEG, 8PEG-acr **(A)**, or 8PEG-vs. **(B)**, macromonomers could react with ammonia via amine-Michael type addition to create 8PEG-olefin hydrogels, which could be modified via sulfur-Michael type addition to generate 8PEG-SH hydrogels (Ouyang, 2018).

The mechanical properties of PEG hydrogels can be varied, depending on the molecular weight, crosslinking density, and synthetic procedure. Besides the tunable elastic property, multiple functionalities to PEG-based hydrogels can be also applied. In addition, star shaped or branched 6- or 8-arm PEG derivatives with the appropriate end groups could be polymerized by photo-polymerization and also by using Michael type addition reactions (Zhang et al., 2014). Using the latter approach, an ability for multifunctional PEG hydrogels is achieved, where certain amounts of acrylate groups are crosslinked and the others are left non-crosslinked for different types of additional multiple functionalities. Thus, various charged or non-charged functional chemical groups can be anchored (Figure 1). Not only molecules but also inorganic nanoparticles provide assorted amounts of functions. Especially gold nanoparticles (Au NPs), which have unique size- and shape -dependent optical properties via surface plasmons, little toxicity, easy synthesis procedures, are desired materials not only for industry, and catalysis but also for biology and medicine (Ren et al., 2017a,b; Yesildag et al., 2017a, 2018a,b).

For providing cell adhesion on the bio-inert PEG surfaces, several micro-fabrication techniques have been developed to modify the surface properties and to promote cellular adhesion; for example, applying patterns of surface nano- or micro-topographies, patterns of elasticities, or chemical modifications. Micro-fabrication techniques can be basically categorized into two classes; the “top-down” and the “bottom-up” approaches. Top-down approaches made by miniaturization of bigger elements, such as photo-lithography, micro-contact printing, micro-molding in capillaries (Kim et al., 1996), fill-molding in capillaries (Kelleher et al., 2012), micro-contact deprinting (Chen et al., 2009), and also electron-beam lithography (van Dorp et al., 2000) are some examples. Examples for bottom-up approaches are self-assembled monolayers (SAM); spin-coating, dip-coating, dip-pen lithography (Piner et al., 1999), block copolymer micelle nanolithography (Glass et al., 2003) or supramolecular self-assembly (House et al., 2009). Reactive micro-contact printing (reactive-μ-CP) is a combination of top-down and bottom-up approaches and is a fast and relatively simple process for patterning of large area on a surface in a short period of time. Hereby the well-known micro-contact printing process (μ-CP) which was developed by Whitesides (Tien et al., 1998) group and the affinity contact printing (α-CP) by Delamarche et al. (Renault et al., 2002) were used as a basis. For the conventional μ-CP process usually a PDMS-stamp, an ink solution and a substrate are required; first of all the stamp is inked and after contacting of the hydrogel with the stamp the ink is transferred onto the surface of the substrate. Whitesides et al. further developed this process and introduced the reactive μ-CP process, where the substrate was made reactive toward the ink molecules, so that a covalent binding among the substrate and ink-molecules could be formed (Yan et al., 1998). In the procedure of Delamarche et al. proteins were printed on surfaces via specific functionalization of stamps with protein-anchoring groups (Renault et al., 2002).

In this work we introduce a novel reactive-μCP process where the reactivity of the surface of the stamp is additionally tuned toward the Au NPs. For this process, first of all, a PDMS-stamp was molded a micro- relief-patterned silicon master as template. As shown in our previous publications, amino-silanized surfaces can be effectively covered with citrate capped Au NPs with high density (Ren et al., 2017a,b; Yesildag et al., 2018b). Hereby the Au NPs were interacting electrostatically with the amino-silane surface and they could be effectively transferred onto PEG hydrogel surfaces by various methods, which we recently published (Ren et al., 2017a,b; Yesildag et al., 2017a,b, 2018a,b).

In this work a novel reactive micro-contact printing approach for patterning of gold nanoparticles (Au NPs) on poly(ethylene glycol) (PEG)-based hydrogels is presented. In this reactive μ-CP method the surface of the micro-relief patterned PDMS-stamp is first functionalized with a self-assembled monolayer (SAM) of amino-silane, exhibiting amino groups at the surface that have affinity for Au NPs, and subsequently decorated with citrate-capped Au NPs via relatively weak, yet collective, electrostatic interactions. The stamp is afterwards brought in conformal contact with flat, functionalized PEG- hydrogels, and the Au NPs are transferred onto the surfaces of these hydrogels. PEG-based hydrogels are on the one hand made of short linear chained PEG-precursors which result in relativelyrigid hydrogels and on the other hand of 8-arm star shaped PEG-precursors which yield softer and multi-functional hydrogels. On these micro-patterned Au NPs—PEG-hydrogel—composites, mouse fibroblast L929 cell adhesion was investigated. Our recent studies have shown effective cell adhesion on Au NPs layered surfaces even without prior bio-functionalization of the Au NPs with specific proteins; proteins included in the cell culture media were sufficient for aiding cells to guide and adhere on the Au NPs patterns. Cells adhere only on Au NPs micro-patterns and avoid the anti-fouling PEG material.

## Materials and Methods

### Preparation of PEG Hydrogels

#### Photo-Polymerization

For the UV-photo-polymerization process acrylated PEG macromonomers were used. Linear PEG-diacrylate was purchased from Sigma Aldrich and used without further modification and the purchased 8-arm PEG macromonomer had hydroxyl-end groups and was acrylated which is briefly described in reference (Zhang, 2015). These PEG macromonomers with acrylate end-groups (PEG diacrylate (*M*w = 575 g/mol) or 8-arm PEG acrylate) were crosslinked using the UV-photo-crosslinking process. For the UV-photo-crosslinking process the liquid PEG diacrylate or the at 60°C molten 8PEG acrylate was mixed with 1% of photo-initiator (Irgacure 2959). For having a good distribution of the photo-initiator the mixture was sonicated for around 5 min. Then the mixture was dispensed on a glass slide and covered with a thin glass cover slip to achieve a flat hydrogel sample. This liquid mixture was put under UV-light source for 8 min and the glass cover slip was peeled off. The result was a thin, flat hydrogel sample, as a stand-alone films.

### Crosslinking Via Michael-Type Addition Reaction

20 wt% of ammonium solution (30% NH_3_ in H_2_O) were added to the precursor solution of 8-arm poly(ethylene glycol) vinyl sulfone (8PEG-VS) with 50 wt% water content at room-temperature under vigorous magnetic stirring until the solution turned to a viscous liquid. The resulting liquids were deposited on a glass slide and covered with a glass cover slip. After 30 min, the 8PEG-VS hydrogels were formed. After gel formation, the transparent polymeric films formed with 5% NH_3_ were peeled off mechanically. The stand-alone films (250–300 μm in thickness) were immersed in DTT solution (5 mg/mL) for 60 min. Afterwards, these hydrogels were washed thoroughly with water for several times and stored in water before use (Ren et al., 2017b).

### Synthesis of Gold Nanoparticles (Au NPs)

The Au NPs were synthesized following the protocol of Bastús et al. (2011) First of all, Au NPs seeds were synthesized. In the next step the seeds were continuously grown to bigger particles; in this work Au NPs with 25 nm sizes are used. For the seed synthesis 150 ml aqueous solution of trisodium citrate (Na_3_C_6_H_5_O_7_) (2.2 mM) (Sigma Aldrich, Germany) was boiled for 15 min. Then 1 ml of HAuCl_4_-solution (25 mM) (Sigma Aldrich, Germany) is injected at once. The color of the solution was changed from yellow to bluish-gray and then finally to soft pink within 10 min. For growing of bigger sized particles the solution was cooled down to a temperature of 90°C. Into that solution 1 ml sodium citrate (60 mM) and 1 ml of a HAuCl_4_-solution (25 mM) was injected. After 30 min the reaction was completed and again 1 ml of a HAuCl_4_-solution (25 mM) was added. Thirty minutes later the solution was diluted by taking out 27.7 ml of the Au NP solution and adding 27.6 ml of water.

### Preparation of Silicon Masters

**Figure 2:**
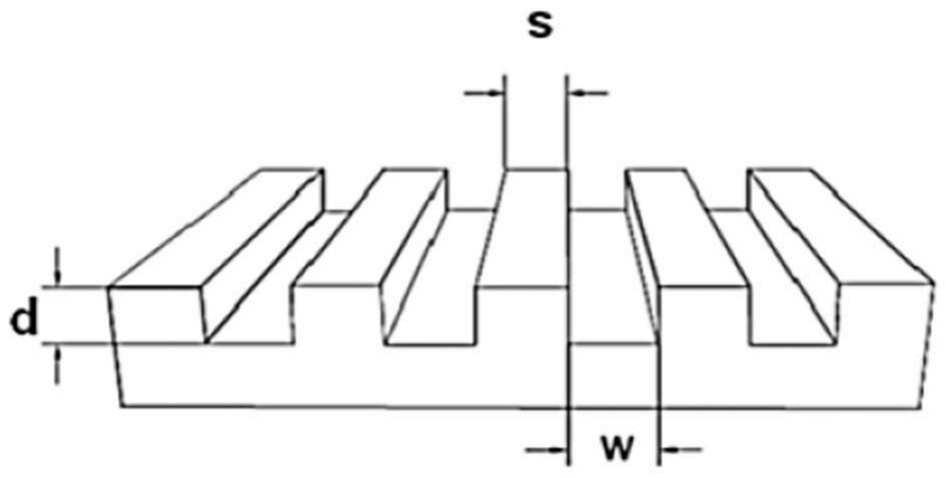
Structure of a silicon master.

For the preparation of the stamps a micro relief patterned silicon master was necessary. The sizes of the masters are described with a three-numeric code: w-s-d = width of the grooves—spacing between the grooves—depth of the grooves, as shown in Figure 2.

The surfaces of the silicon masters were made inert by applying trichloro(1H, 1H, 2H, 2H-perfluorooctyl)silane (Sigma Aldrich) molecules on its surface. For that the silicon masters were cleaned with water, acetone, and isopropanol and dried with a stream of nitrogen. For the activation of the surface, the cleaned silicon masters were oxidized via immersion in a piranha solution (H_2_SO_4_: H_2_O_2_; 7: 3; v/v) for 30 min. Then the master was washed with deionized water and isopropanol and dried with a stream of nitrogen. After the activation of the surfaces, the silicon masters were placed in a clean petridish and then placed into a desiccator. Incidentally, in a small vial containing 1–2 drops of the silanizing agent perfluoro-silane agent was placed into the desiccator together with the silicon masters. Then, the desiccator was kept under vacuum for 2 h. After that, the silanized silicon wafers were washed with toluene and isopropanol and then dried under a flow of nitrogen.

### Preparation of Au NPs Decorated PDMS-Stamps

The inert silicon master was cleaned with water, acetone and isopropanol and dried with a stream of nitrogen and is used as tamplate for the PDMS-stamp.

**Figure 3:**
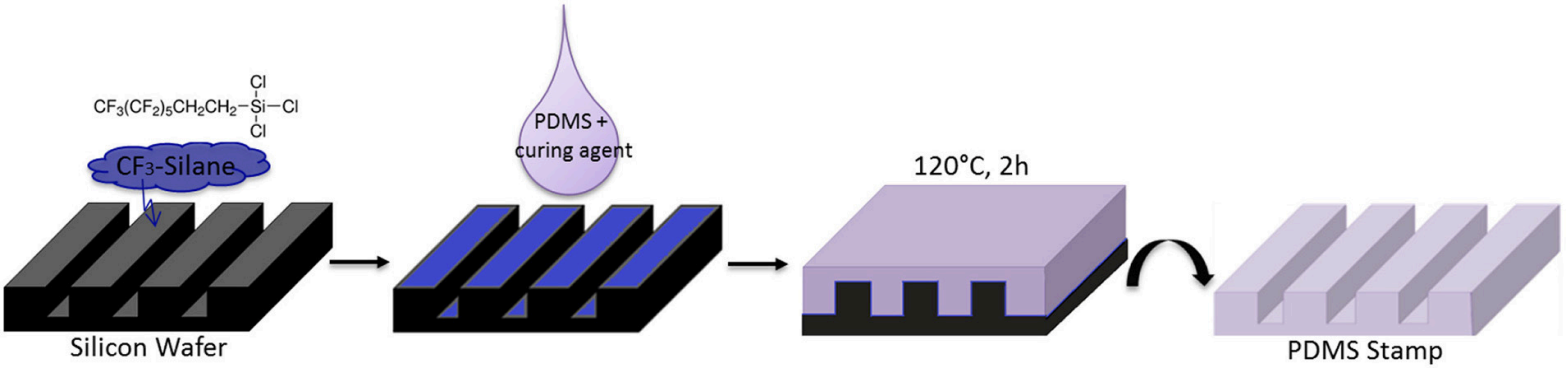
Schematic view of PDMS-Stamp preparation on perfluoro-silanized silicon wafers.

The PDMS-stamp was prepared by using a mixture of Sylgard 184 silicone elastomer and curing agent (10:1; v/v). Then the mixture was degassed in a desiccator and casted on the inert silicon master using the micromolding process, cured for 2 h at 120°C and finally was peeled off from the silicon master (see Figure 3).

**Figure 4:**
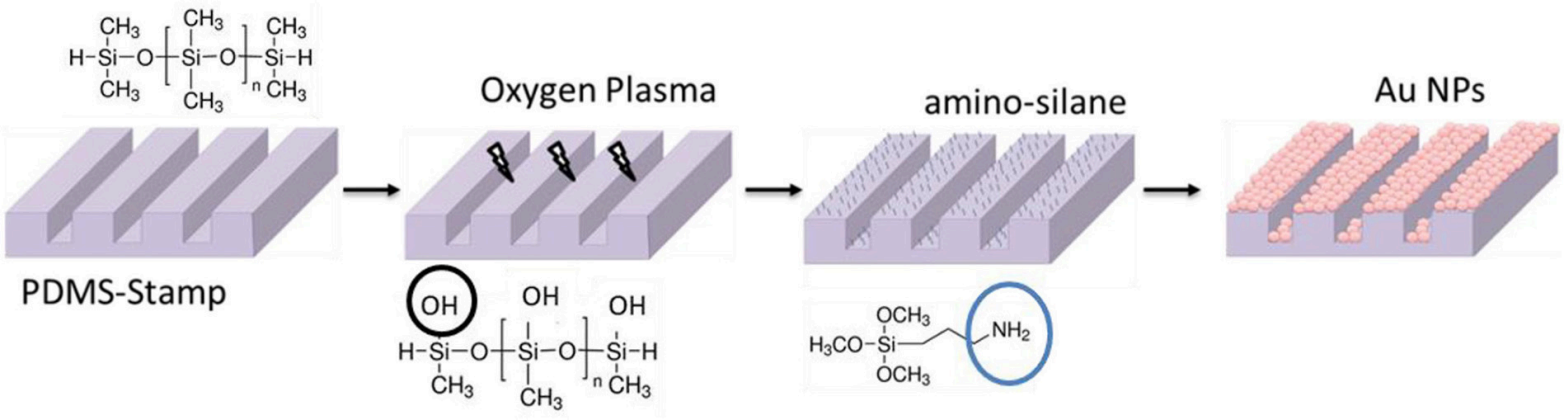
Schematic view of amino-silanization of PDMS-stamps and coating with Au NPs.

The as-prepared PDMS-stamps were oxidized in oxygen plasma for around 10 min. Thereafter the oxidized PDMS-stamps were silanized with aminopropyltrimethoxysilane (Sigma Aldrich) in a desiccator using the vapor method; for that the PDMS-stamps were placed in a desiccator containing two drops of amino-silane agent and vacuum was kept for 2 h. After the amino-silanization of the PDMS-stamps they were immersed in Au NPs solutions for around 1 h (see Figure 4). Thereafter the stamps containing the Au NPs on their surfaces were washed with water and dried under a stream of nitrogen gas.

### Reactive Micro-Contact Printing on PEG Hydrogels

**Figure 5:**
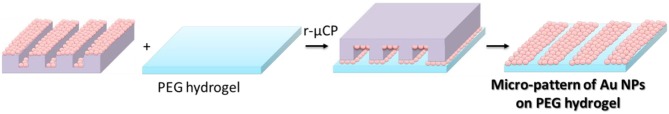
Schematic view of reactive micro-contact printing (r-μCP) process of Au NPs on PEG hydrogels.

**Figure 6:**
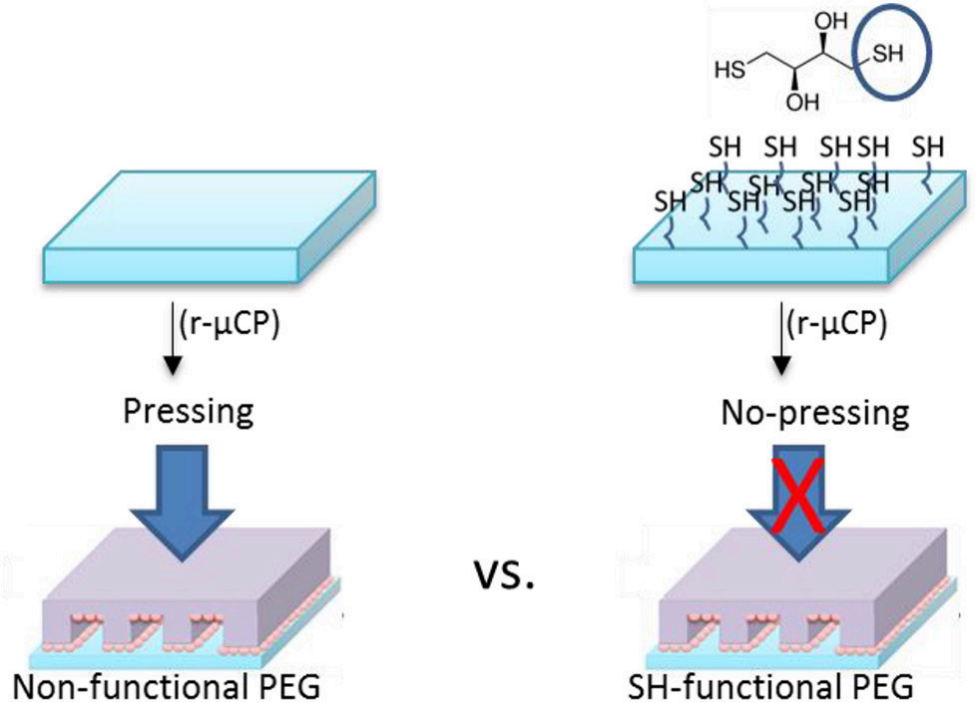
Reactive micro-contact printing (r-μCP) on Non-functional vs. SH-functional PEG.

For the reactive micro-contact printing process, the free standing PEG hydrogels were prepared following the procedures described above. These hydrogels were then contacted with the Au NPs coated PDMS-stamps (see Figure 5). Depending on the reactivity of the PEG hydrogel the contact force is was varied: on the hard and rigid PEG hydrogel the stamp was firmly contacted with the PEG hydrogel in order to transfer the Au NPs from the stamp onto the surface of hydrogels. For multifunctional hydrogels from 8PEG precursors with thiol surface functions the hydrogel was contacted only with a mere contact and the Au NPs were readily transferred onto the surface of the hydrogel through aided by strong and collective chemical interactions (see Figure 6).

### Cell Adhesion

Mouse fibroblasts L929 cells were cultured in the tissue culture plate in RPMI 1,640 medium with addition of 10% Fetal Bovine Serum (FBS) and 1% Penicillin/Streptomycin (PS) in a cell culture plate in an incubator at controlled temperature (37°C) and CO_2_ atmosphere (5%).

First of all a confluency of at least 75% should be reached. Thereafter the cells were washed with PBS, detached by using trypsin and after the centrifugation process a new medium is added on the cells and mixed properly. Ten microliter of this cell medium solution is put on a cell counter chamber in order to count the cell number by using an optical microscope and achieve a concentration of 40,000 cells/ml. Depending on the counted cell number the cell solution was mixed with a defined amount of new medium. The samples were placed in a TCPS plate; two drops of Au NPs solution or the washed and precut hydrogel. The samples were then cultured within these cells for 24 h, at 37°C in a 5% CO_2_ atmosphere.

### Critical Point Drying

Critical point drying is an established method of dehydrating biological tissues before the scanning electron microscopic (SEM) imaging (Rostgaard and Christensen, 1975)^1^. In the course of this work murine fibroblasts, which adhered on specifically designed surfaces were inter alia measured by SEM. For that after 24 h of incubation the cells were prior fixed on the sample on the sample using 4% formaldehyde for 30 min. Then the medium is removed and replaced by PBS, which was later stepwise exchanged by ethanol or acetone (30–100% v/v) and the samples were placed in a critical point drying apparatus, where ethanol or acetone was exchanged by CO_2_. The final dried samples were sputtered with carbon.

### Characterization Instruments

Optical images were taken with the Axio Oberserver.Z1 (Carl Zeiss) and analyzed using the Axio Vision software (V4.8.2 Carl Zeiss). Scanning electron images were taken with a Hitachi S-520 using an acceleration voltage of 20 kV and a working distance of 10 mm. Pictures were taken using the Digital Image Processing System(2.6.20.1, Point Electronic).

## Results and Discussion

First of all PDMS-stamps were prepared by using a perfluorinated silicon master that had topographical sizes of [w-s-d] = width of the grooves—spacing between the grooves—depth of the grooves of a relief pattern: [20–10–5 μm] (see Figure 3).

**Figure 7:**
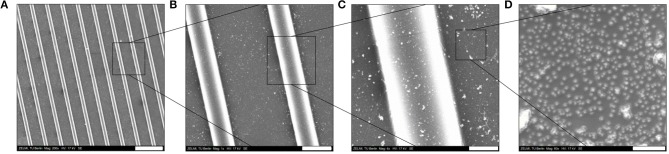
SEM images of amino-silanized PDMS-stamp (used master sizes was [20–10–5 μm]) inked with Au NPs: **(A)** overview of the stamp surface; **(B)** enlarged view of the topographical line; **(C)** enlarged view of the groove; **(D)** Au NPs distribution on the stamp surface. Scale bars: (A) 50 μm; **(B)** 10 μm; **(C)** 3 μm; **(D)** 200 nm.

Thereafter, as schematically shown in Figure 4, the surface of the PDMS-stamp was activated by oxidation in oxygen plasma for around 10 min and silanized with amino-silane using the vapor silanization method. Thereafter, the PDMS-stamp was inked with Au NPs solution for 1 h and washed with water and dried with a stream of nitrogen. The Au NPs-coated PDMS-stamp is shown in the SEM images in Figures 7A–D.

In Figures 7B,C it can be observed that the Au NPs were located on the topographical lines and also on the grooves and Figure 7D shows the distribution of the Au NPs on the stamp surface in an enlarged view; there it can be seen that the surface coverage of the Au NPs was relatively high which was also expected due to the electrostatic attractive interactions between the citrate-capped Au NPs and the amino-silane layer. Besides the well-resolved Au NPs there were also larger (around 100–500 nm) white features observables, which might be agglomerated Au NPs and/or uneven PDMS features where the electrons couldn't be conducted and caused a brighter image. The thus Au NPs-inked PDMS-stamps were contacted with PEG hydrogel surfaces; the process was schematically depicted in Figure 5.

**Figure 8:**
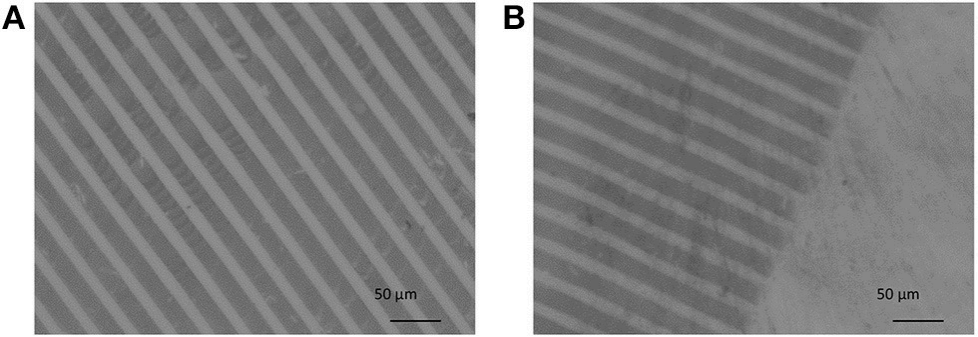
**(A,B)** Optical micrograph of Au NPs on PEG-hydrogel fabricated by the reactive μ-CP process. Scale bars: **(A,B)** 50 μm.

The Au NPs were then successfully transferred onto the surface of the PEG hydrogels, which can be recognized by optical microscopy, as evident from the micrographs in Figure 8.

Depending on the type of PEG-precursors (linear or 8PEG), and availability of reactive functional groups on PEG-based hydrogel surfaces, the transfer efficiency could be optimized, which we have shown in our previous publications (Ren et al., 2017a,b). The highest transfer yield was achieved on thiol-functionalized PEG hydrogel derivatives. The interaction of amino-silane surface among the PDMS-stamp and the Au NPs were relatively weak electrostatic interactions which can be quite easily released; also onto non-functional PEG hydrogels, Au NPs could be transferred with applying certain forces. On the other hand, the interaction of the Au NPs with the mercapto/thiol-functionalised PEG hydrogels is strong; in that case, even without applying any forces, i.e., just by slight contact, transfer of the Au NPs pattern was effective. For this process, the interactions or reactivity of all surfaces—stamp and PEG hydrogel—with the Au NPs was precisely tuned.

In Figure 8 the darker lines represent the Au NPs micro-pattern and the brighter area the non-contacted, pure PEG hydrogel. As shown in Figure 7, the contact line pattern area of the stamp had widths of 20 μm and distances of 10 μm. That can be also seen in Figures 8A,B. Taking a closer look into the micrograph in Figure 8, some white spots are visible. In Figure 8B the edge of the contact area of the stamp with the PEG hydrogel is seen. Nearly the original pattern size of the PDMS-stamp was clearly printed on PEG hydrogel without showing notable defect structures.

**Figure 9:**
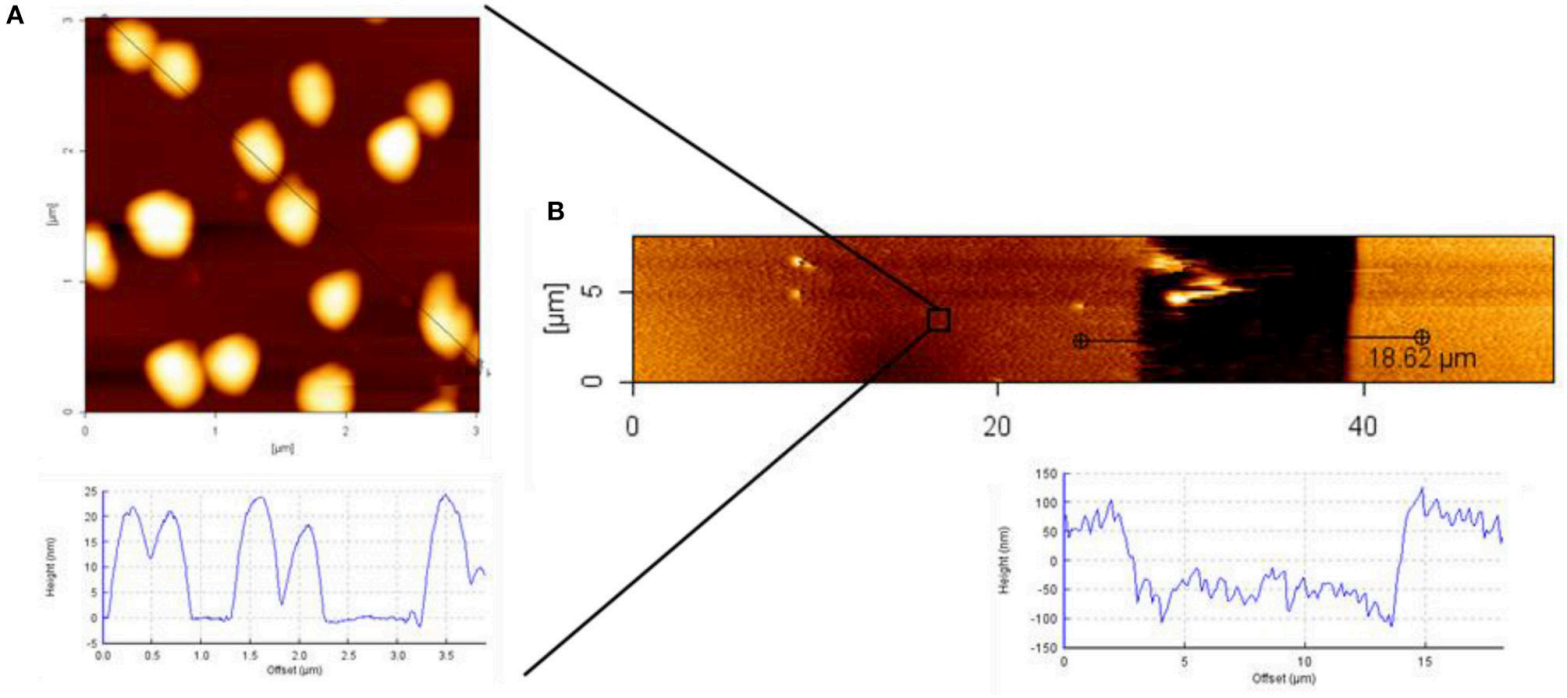
AFM height image and cross section profile of: **(A)** Au NPs on silicon wafer; **(B)** Au NPs pattern on PEG hydrogel; used stamp size [15-50-15].

For an even more detailed characterization of the transferred patterns of Au NPs, atomic force microscopy (AFM) measurements were conducted. In Figure, the AFM characterization of the Au NPs pattern on 8PEG hydrogel is shown. The size of the particles can be recognized from Figure 9A; the sizes of the particles were around 20 nm. In Figure 9B the pattern is shown, hereby the stamp had sizes of [15-50-15], thus the stamped area was 50 μm and the gap between the contacts were 15 μm, which is clearly observable in the AFM image.

After having achieved a clear and documented micro-pattern of Au NPs on PEG hydrogel surfaces, cell adhesion study was investigated. Hereby murine fibroblast L929 cells were cultured on the Au NPs patterned PEG hydrogels in a cell culture medium, which contained 1% PS and 10% FBS, in a 5% CO_2_ atmosphere at 37°C for 24 h.

**Figure 10:**
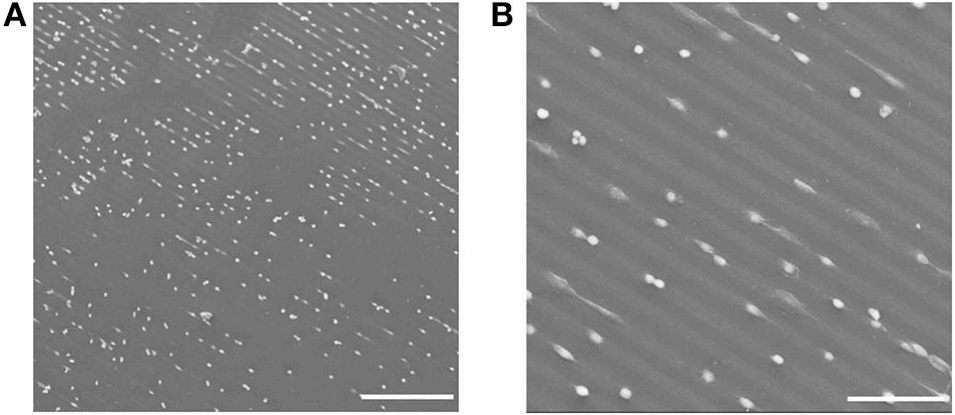
**(A,B)** SEM images of cell adhesion on mico-patterned Au NPs lines on PEG-hydrogel. Scale bars: **(A)** 300 μm; **(B)** 100 μm.

In Figures 10A,B SEM images of the cell adhesion results are shown. In order to be able to image cells with SEM without losing the original structure, the cells were fixed with formaldehyde on the hydrogel for 30 min and dried via critical point drying procedure. After sputtering the hydrogels with carbon the SEM measurement was performed. In Figures 10A,B it can be seen that the cells were adhering on the Au NPs patterned PEG hydrogel surface; following the patterned Au NPs lines.

**Figure 11:**
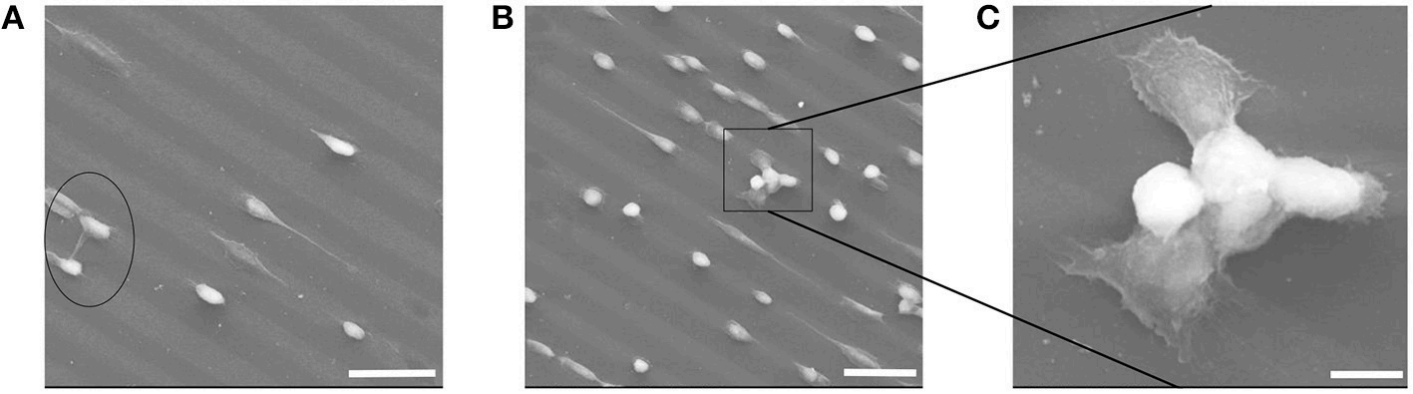
**(A–C)** SEM images of cell adhesion on Au NPs micro-patterns exhibiting line-bridging cells. Scale bars: (A) 50 μm; **(B)** 50 μm; **(C)** 10 μm.

PEG materials are known to be inert to biomolecular interactions. That was also observable on the micro-patterned PEG Au NPs surfaces; the cells effectively avoided the pure PEG hydrogel and only adhered on Au NPs layers. The hereby chosen pattern sizes of 20 μm for the Au NPs lines and 10 μm for the distances between the Au NPs lines allowed cells bridging over the PEG lines and to have contacts with two parallel Au NPs lines. This was observable in a few cases, such as in Figure 11. In Figure 11A the bridge-over of one whole cell happened via stretching of protrusions, while in Figures 11B,C the bridging occurred via joining of a few cells. Figure 11C is an enlarged view of the joined bridging cells; on this SEM image it can be obviously seen that the cells that had contacts with the Au NPs lines were spread and show adherent protrusions, while on the bridging area over the PEG hydrogel line no protrusions were visible; that means that they didn't have any adhesive contacts with this line.

**Figure 12:**
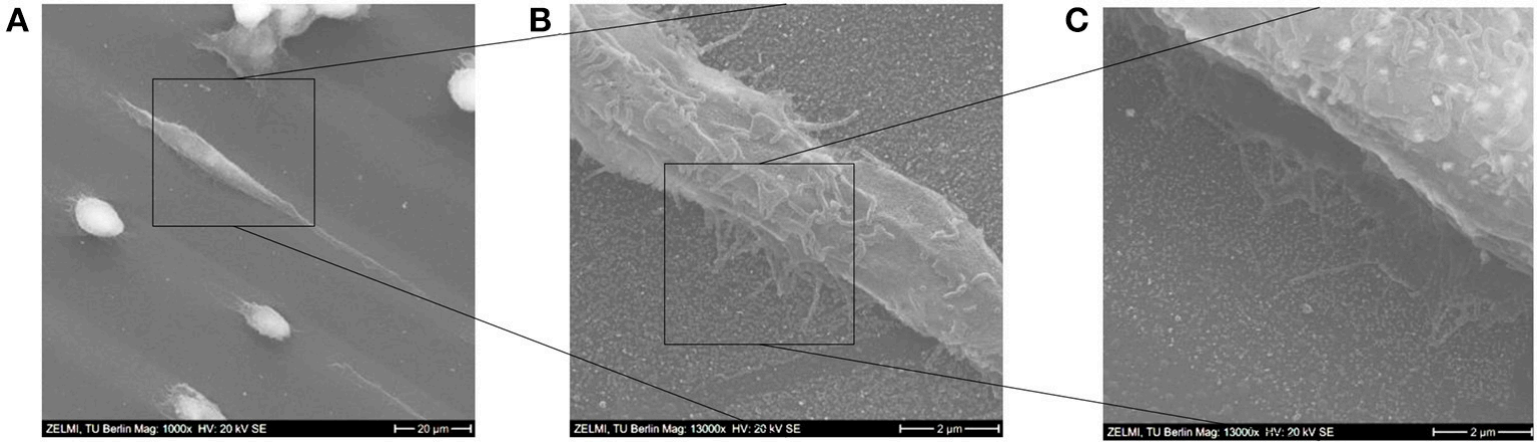
**(A–C)** SEM images of a stretched cell on the Au NPs micro-pattern. Scale bars: **(A)** 20 μm; **(B)** 2 μm; **(C)** 2 μm.

In the SEM images in Figure 12A the morphologies of some adhered cells are shown; while some cells were adherent, yet only stretched to around 110–150% of their original sizes, other ones were elongated, and had lengths of around 300% of their original sizes. After the cells were settled down they started forming focal adhesions with their lamellipodia and show also some finger-like protrusions, which is more clearly visible in the SEM images in Figures 12B,C.

**Figure 13:**
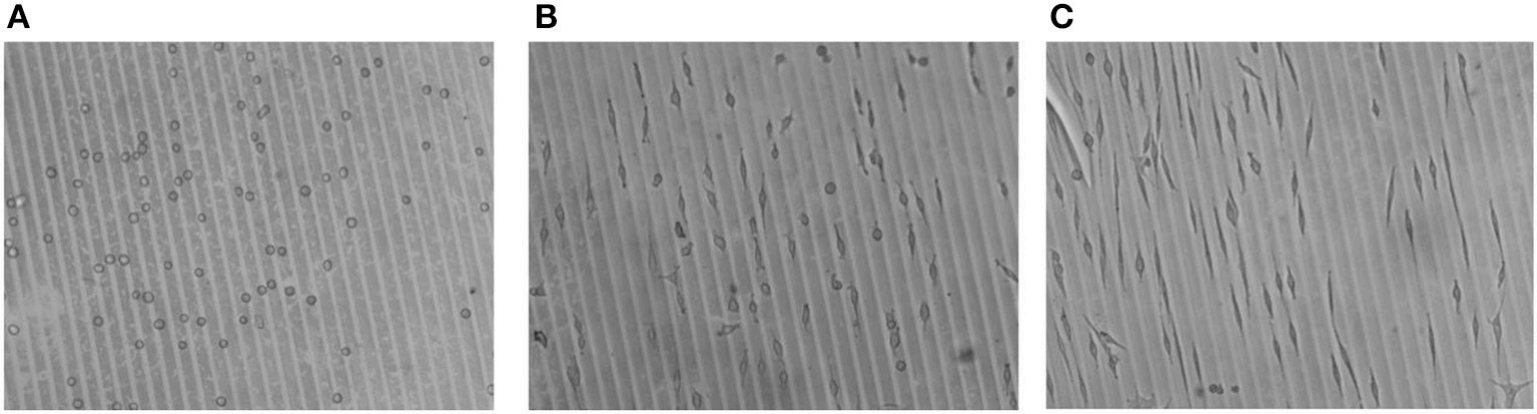
Optical micrographs of cell adhesion on Au NPs patterned 8PEG hydrogel : **(A)** directly after seeding (0 min); **(B)** after 24 h, and **(C)** after 48h of cell culture.

In Figure 13, a larger area of cell culture on the micro-patterned hydrogels is shown, to give the overall impression of selective cell adhesion to the micro-lines of Au NPs and adaptation of the cell morphology, i.e., elongation.

As a subconclusion, it can be said that micro-patterning with Au NPs on PEG based hydrogels was an effective way in order to guide the cells to follow specific paths and have adhesions only on these areas while avoiding unwanted areas; hence the cell behavior could be effectively controlled.

**Figure 14:**
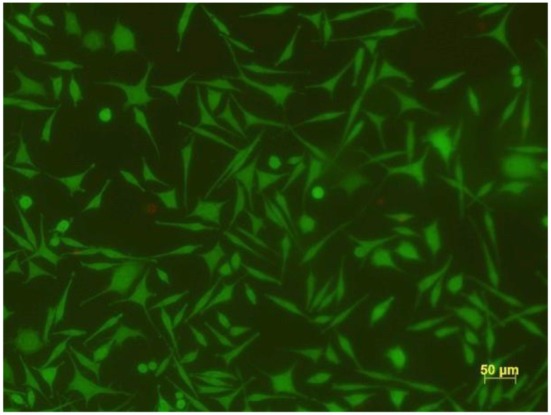
Live/dead assay on Au NPs decorated 8PEG hydrogel surface.

Finally, and not less importantly, the viability of the cells on these novel nanocomposite materials was investigated. In Figure 14 the result of the live/dead assay is shown, manifesting that at least 99% of the cells were alive.

## Conclusions

In this work a novel reactive micro-contact printing approach for patterning of gold nanoparticles (Au NPs) on poly(ethylene glycol) (PEG)-based hydrogels was presented. For this patterning method, the surface of the micro-relief patterned PDMS-stamp was functionalized with an amino-silane self-assembled monolayer (SAM) and was subsequently decorated with citrate capped Au NPs via electrostatic interactions. The stamp was afterwards brought in conformal contact with flat PEG-based hydrogels and the Au NPs were transferred onto the surfaces of these functional hydrogels. Depending on the chemical functionality on PEG hydrogel surfaces the transfer could be facilitated. Stamping on non-functional PEG hydrogel surfaces required certain force in order to transfer the Au NPs onto its surface, while on specifically functionalized PEG hydrogel surface with thiol-functions only a slight contact was sufficient to transfer the Au NPs efficiently onto the surface of PEG hydrogels. On these micro-patterned Au NPs—PEG-hydrogel—composite biointerfaces, murine fibroblast L929 cell adhesion was investigated. Cells adhered only on Au NPs micro-patterns and effectively avoided the anti-adhesive PEG background. With this method, a platform to control the cell adhesion has been established. Furthermore, owing to the unique optical characteristics of the Au NPs, these nanocomposite materials are potentially useful probes for SERS or LSPR applications.

## Author Contributions

CY and ZZ conceived and performed the experiments and analyzed the data. ZO synthesized the macromonomers and CY wrote the paper. ML monitored and guided the process of designing the experiments, contributing the methods and infrastructure, interpreting and discussing the data, and improving the manuscript.

### Conflict of Interest Statement

The authors declare that the research was conducted in the absence of any commercial or financial relationships that could be construed as a potential conflict of interest.

## Footnotes

^2^https://www.emsdiasum.com/microscopy/technical/datasheet/critical_drying.aspx
